# Knockdown of AGGF1 inhibits the invasion and migration of gastric cancer via epithelial–mesenchymal transition through Wnt/β-catenin pathway

**DOI:** 10.1186/s12935-019-0765-6

**Published:** 2019-02-27

**Authors:** Han-Hui Yao, Ya-Jun Zhao, Yi-Fu He, Da-Bing Huang, Wei Wang

**Affiliations:** 1 Department of General Surgery, The First Affiliated Hospital of USTC, Division of Life Sciences and Medicine, University of Science and Technology of China, Hefei, Anhui, 230001 P.R. China; 2 Department of Medical Oncology, The First Affiliated Hospital of USTC, Division of Life Sciences and Medicine, University of Science and Technology of China, No. 17 Lujiang Road, Hefei, Anhui, 230001 P.R. China

**Keywords:** Gastric cancer, AGGF1, Epithelial mesenchymal transition, Wnt/β-catenin pathway, Invasion and migration

## Abstract

**Background:**

Angiogenic factor with G-patch and FHA domain 1 (AGGF1), as a newly identified human angiogenic factor, is overexpressed in some types of malignant tumors and closely associated with patient’s prognosis. However, the mechanisms involved in the regulation of AGGF1 in gastric cancer (GC) still remain unclear.

**Methods:**

In this study, AGGF1 level in GC tissues and cell lines was analyzed by western blot and quantitative real-time polymerase chain reaction (qRT-PCR). After knockdown of AGGF expression by RNA interference in GC cell lines MKN-45 and MGC-803, wound healing and transwell assays were conducted to examine the effects of AGGF1 on migration and invasion. Tumor growth was assessed in a mouse xenograft model in vivo. Furthermore, expression levels of epithelial–mesenchymal transition (EMT) biomarkers and involvement of the Wnt/β-catenin pathway were detected by western blot and qRT-PCR.

**Results:**

Compared to those in normal groups, the protein and mRNA of AGGF1 expression levels were significantly higher both in GC tissues and cell lines (all P < 0.05). Knockdown of AGGF1 dramatically inhibited the invasion and migration of MKN-45 and MGC-803 cells (all P < 0.01) in vitro, and suppressed the tumor growth of nude mice xenograft model in vivo. Western blot revealed alterations in EMT biomarkers, suggesting the role of AGGF1 in EMT. Moreover, we found that downregulated expression of AGGF1 attenuated Wnt/β-catenin related protein expression.

**Conclusions:**

Collectively, knockdown of AGGF1 inhibits the invasion and migration of gastric cancer via epithelial–mesenchymal transition through Wnt/β-catenin pathway.

**Electronic supplementary material:**

The online version of this article (10.1186/s12935-019-0765-6) contains supplementary material, which is available to authorized users.

## Background

Gastric cancer (GC) is one of the most common malignant gastrointestinal tumors in the world [1, 2]. Although its mortality has decreased significantly over the past 20 years, its morbidity and mortality are still at the forefront of malignant tumors in China [3, 4]. Obviously, the major obstacle for GC treatment failure is tumor metastasis, during which invasion and migration are the pivotal steps. In recent years, epithelial–mesenchymal transition (EMT) has become a research hotspot of tumor metastasis. EMT is a key process during embryonic morphogenesis, heart development, wound healing, and cancer metastasis [5, 6]. During EMT, epithelial cells lose their junctions and apical-basal polarity, reorganize their cytoskeleton and undergo a change in the signaling programmes. This ultimately increases the motility of individual cells and enables the development of an invasive phenotype. Therefore, through exploration of the molecular mechanism of EMT in GC, it not only guide a new research direction for the biological behavior of GC metastasis, but also provide a potential strategy for the treatment of GC.

Angiogenic factor with G-patch and FHA domain 1 (AGGF1 or VG5Q), as a newly identified human angiogenic factor, was first reported by Tian et al. [7] in 2004. Recent studies have found that AGGF1 is expressed in some types of malignant tumors and is closely related to tumor angiogenesis [8–11]. Besides, our previous study has revealed that AGGF1 expression was significantly associated with the lymph node metastasis, invasion depth and TNM stage of GC patients [12]. Moreover, high expression of AGGF1 could be used as an independent factor to predict poor postoperative survival of GC patients [12]. However, the detailed regulatory mechanism of AGGF1 in the invasion and metastasis of GC still remains unclear. Interestingly, Major et al. [13] have identified the new regulators of Wnt/β-catenin signaling by using integrative molecular screening and characterized AGGF1 as a nuclear chromatin-associated protein that participates in β-catenin-mediated transcription in human colon cancer cells. Moreover, Wnt/β-catenin signaling is one of the most important signaling pathways involved in EMT of malignancies including GC [14–16]. Therefore, the issue whether AGGF1 can regulate the EMT of GC through Wnt/β-catenin signaling has attracted our great interest and concern.

In this study, we used in vitro and in vivo approaches to demonstrate that whether knockdown of AGGF1 could inhibit EMT and whether the regulatory effects of AGGF1 on the EMT were partially attributed to the Wnt/β-catenin signaling pathway in GC.

## Methods

### Clinical specimens

Forty cases of fresh gastric cancer samples and adjacent noncancerous tissues were collected from patients that underwent curative gastric cancer resection at the Department of General Surgery in our hospital. Samples were dissected from resected specimens by a pathologist, and immediately snap-frozen in individual vials using liquid nitrogen. Frozen specimens were stored at − 70 °C in a tumor bank until further AGGF1 expression detection by western blot and qRT-PCR. Written consent was obtained from all patients, and all experiments were performed in accordance with The Code of Ethics of the World Medical Association (Declaration of Helsinki). The study (including the collection and use of patients’ samples) was approved by the Ethics Committee of the First Affiliated Hospital of USTC.

### Cell culture

Four human gastric cancer cell lines (SGC-7901, MGC-803, MKN-45 and AGS) and one normal human gastric epithelium cell line (GES-1) were purchased from Oncogene Biotechnology Company (Yangzhou, P. R. China), and cultured in RPMI-1640 or DMEM medium. All cell lines were maintained at 37 °C, 5% CO_2_. The characteristics and origin of GC cell lines used in the study were summarized in Additional file 1: Table S1.

### RNA interference and plasmid construct

The small interfering RNA (siRNA) was used for the knockdown of endogenous AGGF1 in MKN-45 and MGC-803 GC cells. The target sequence was: 5′-CGAATGAAGATCATCAAGAAT-3′, and a non-targeting sequence was used as a normal control (NC). Cells with depleted endogenous AGGF1 expression were selected by being cultured in puromycin at the final selection concentration of 2 μg/mL. To construct β-catenin overexpression in GC cells, PCR product covering β-catenin open reading frame (Gene Bank Accession No. NM001012329) was cloned into the pcDNA3.1 vector. MKN-45 GC cells after AGGF1 knockdown were transfected with the β-catenin overexpression vector by Lipofectamine 3000 (Invitrogen, USA) according to manufacturer’s protocol.

### Wound-healing assay

Scratch was made by a pipette tip after the formation of a monolayer of cells. Then, cells were incubated in DMEM medium containing 10% FBS at 37 °C. Gap size was measured at 0 and 24 h later. Besides, the migration distance was calculated at each time point in three independent samples.

### Cell invasion and migration assays

Transwell cell migration assays were performed in 24-well plates with 8.0 µm permeable polycarbonate membrane. Matrigel at high concentration and Growth-factor lowered the Matrigel (BD Biosciences, San Diego, CA, USA) through the spread of each bottom of transwell chamber. Subsequently, cells were diluted by serum-free basic culture medium into 5 × 10^5^/mL, followed by transferring 0.5–1 mL to each transwell using pipette guns. Contrarily, we filled the lower wells by culture media with 10% FBS as a chemoattractant. Thereafter, the wells were incubated at 37 °C for 24 h in a moistened cell culture incubator. The non-invading cells on the membrane’s top side were removed by scrubbing. The invading cells on the migrated side were fixed in 10% formalin for 10 min, followed by staining with 0.1% crystal violet.

### RNA isolation and qRT-PCR

Total RNA was extracted from the cell lines using TRIzol (Invitrogen, Carlsbad, CA, USA), according to manufacturer’s instructions. The absorbance of RNA was measured at 260 nm using a NanoDrop spectrophotometer (ND-1000, Thermo Scientific, Waltham, MA, USA), in order to determine the total RNA concentration. Reverse transcription of 2 µg total RNA was conducted using the Prime Script RT reagent kit, gDNA Eraser (TaKaRa, Japan). Based on the manufacturer’s protocol, the ABI 7500 fast real-time PCR system (Applied Biosystems, Foster City, CA, USA) together with SYBR Green PCR Master Mix (Applied Biosystems, Foster City, CA, USA) were used with a first step at 95 °C for 10 min followed by 40 cycles with 95 °C for 15 s and 60 °C for 1 min, with a fluorescent reading at the end of this step to amplify the specific genes. The primers used in PCR amplification were listed in Table 1. GAPDH was used as the interval control to calculate the relative expression level of testing genes using the comparative delta Cq (2^−ΔΔCq^) method. Moreover, independent determination of each sample was performed thrice, and the mean value of the expression levels was calculated.

**Table 1:** The primer sequences for PCR used in the study

Genes	Forward (from 5′ to 3′)	Reverse (from 5′ to 3′)
AGGF1	GCATCACACAGAACGGCTGTA	TCATTTCTCCCACGTTGGAGTAT
E-Cadherin	CGAGAGCTACACGTTCACGG	GGGTGTCGAGGGAAAAATAGG
β-Catenin	ACGGAGGAAGGTCTGAGGAG	AGCCGCTTTTCTGTCTGGTT
Snail	TCCAGAGTTTACCTTCCAGCA	CTTTCCCACTGTCCTCATCTG
Vimentin	GGACCAGCTAACCAACGACA	AAGGTCAAGACGTGCCAGAG
GAPDH	CGTCCCGTAGACAAAATGGT	TTGATGGCAACAATCTCCAC

### In vivo tumor xenograft assays

NOD/SCID mice (6–8 weeks old) were purchased from Model Animal Research Center of Nanjing University. A lentiviral shRNA vector targeting AGGF1 was generated by inserting stranded oligonucleotides (shAGGF1, forward sequence 5′-CCGGATGGGTAGTGGAGCCTAATTTCTCGAGAAATTAGGCTCCACTACCCATTTTTTG-3′) into pLKO-puro Vector (Sigma-Aldrich). The cells were infected with AGGF1 shRNA vectors and selected with puromycin (5 μg/mL). Each mouse was injected subcutaneously into the dorsal of the mice with empty vector-transfected cells (4 × 10^6^) or with sh-AGGF1 cells (4 × 10^6^). Mice were euthanized, and the tumors were excised after 40 days. All animal experiments complied with the ARRIVE guidelines and were carried out in accordance with the National Institutes of Health guide for the care and use of Laboratory animals (NIH Publications No. 8023, revised 1978) and the guidelines of the First Affiliated Hospital of USTC.

### Western blotting

All cells were lysed completely in lysis buffer at 4 °C. Then, total protein concentration was calculated using the BCA protein assay kit (Beyotime, Jiangsu, China). To break the structure of protein, the protein was heated at 100 °C for 10 min. Following that, equivalent amount of total protein was added into each well of 10% polyacrylamide gels. Subsequent to electrophoresis, the protein was transferred to nitrocellulose membranes, followed by soaking the same in 5% bovine serum albumin (BSA) or 5% non-fat milk diluted in Tris Buffered Saline Tween (TBST) for 2 h. Thereafter, the blocked membranes were incubated in primary antibodies AGGF1 (1:1000; Proteintech, IL, USA), E-cadherin (1:1500; Cell Signaling Technology Danvers, MA, USA), vimentin (1:1000; Cell Signaling Technology Danvers, MA, USA), snail (1:1000; Cell Signaling Technology Danvers, MA, USA), β-catenin (1:1000; Cell Signaling Technology Danvers, MA, USA), GSK-3β (1:1000; Cell Signaling Technology Danvers, MA, USA), Phosphorylated GSK-3β (1:1000; Cell Signaling Technology Danvers, MA, USA), and GAPDH (1:1000; Abmart, Shanghai, China) at 4 °C overnight. Subsequent to washing the membranes with TBST, the membranes were incubated with a secondary antibody (dilution, 1: 5000; Cat. No. KC-RB-035; KangCheng Bio-tech, Shanghai, China) for a period of approximately 60 min at room temperature. After washing the membranes with TBST, the membranes were ameliorated through the ECL kit (Thermo Scientific), followed by capturing the emitted signals using KODAK X-OMAT BT Film (Kodak, Rochester, NY). The gray value of the protein was measured by performing the ImageJ software (National Institutes of Health, Bethesda, MD, USA).

### Statistical analysis

All data were analyzed using GraphPad Prism 5.0 (GraphPad Software Inc., La Jolla, CA, USA). Two-tailed Student’s t-test was used to determine the differences between groups. P < 0.05 was considered statistically significant.

## Results

### AGGF1 was upregulated in GC tissue samples and cell lines

**Fig. 1:**
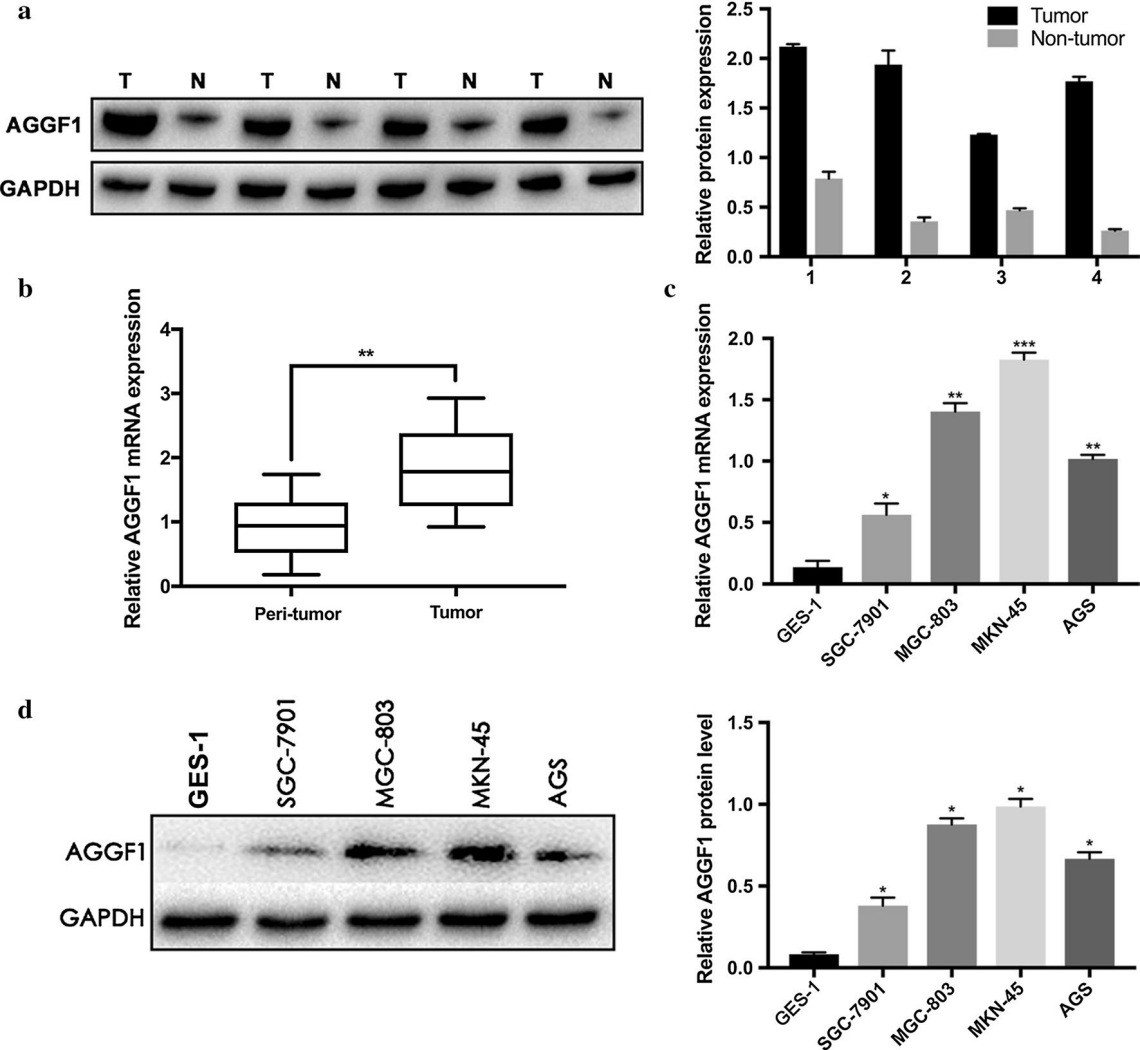
High expression of AGGF1 in GC tissues and cell lines. The protein (**a**) and mRNA (**b**) levels of AGGF1 in GC and adjacent normal tissues. The mRNA (**c**) and protein (**d**) levels of AGGF1 in normal gastric mucous epithelium cell (GES-1) and four GC cells (SGC-7901, MGC-803, MKN-45 and AGS). Data were presented as mean ± SD. GAPDH served as an internal reference. All experiments were performed three times. *P < 0.05, **P < 0.01, ***P < 0.001

Initially, the protein and mRNA levels of AGGF1 in 40 pairs of frozen GC and corresponding normal tissues were detected by western blot and qRT-PCR, respectively. As shown in Fig. 1a, AGGF1 protein levels were upregulated in GC tissues compared with the matched adjacent normal tissues (P < 0.001). Besides, AGGF1 mRNA levels were increased in GC tissues compared with the matched adjacent normal tissues (P < 0.01, Fig. 1b). Then, AGGF1 expression levels in 4 GC cell lines (SGC-7901, MGC-803, MKN-45 and AGS) and GES-1 (normal control) were examined. The results showed that both mRNA and protein levels of AGGF1 were upregulated in GC cell lines compared with GES-1 (all P < 0.05, Fig. 1c, d).

### Knockdown of AGGF1 inhibited the migration and invasion of GC cells

**Fig. 2:**
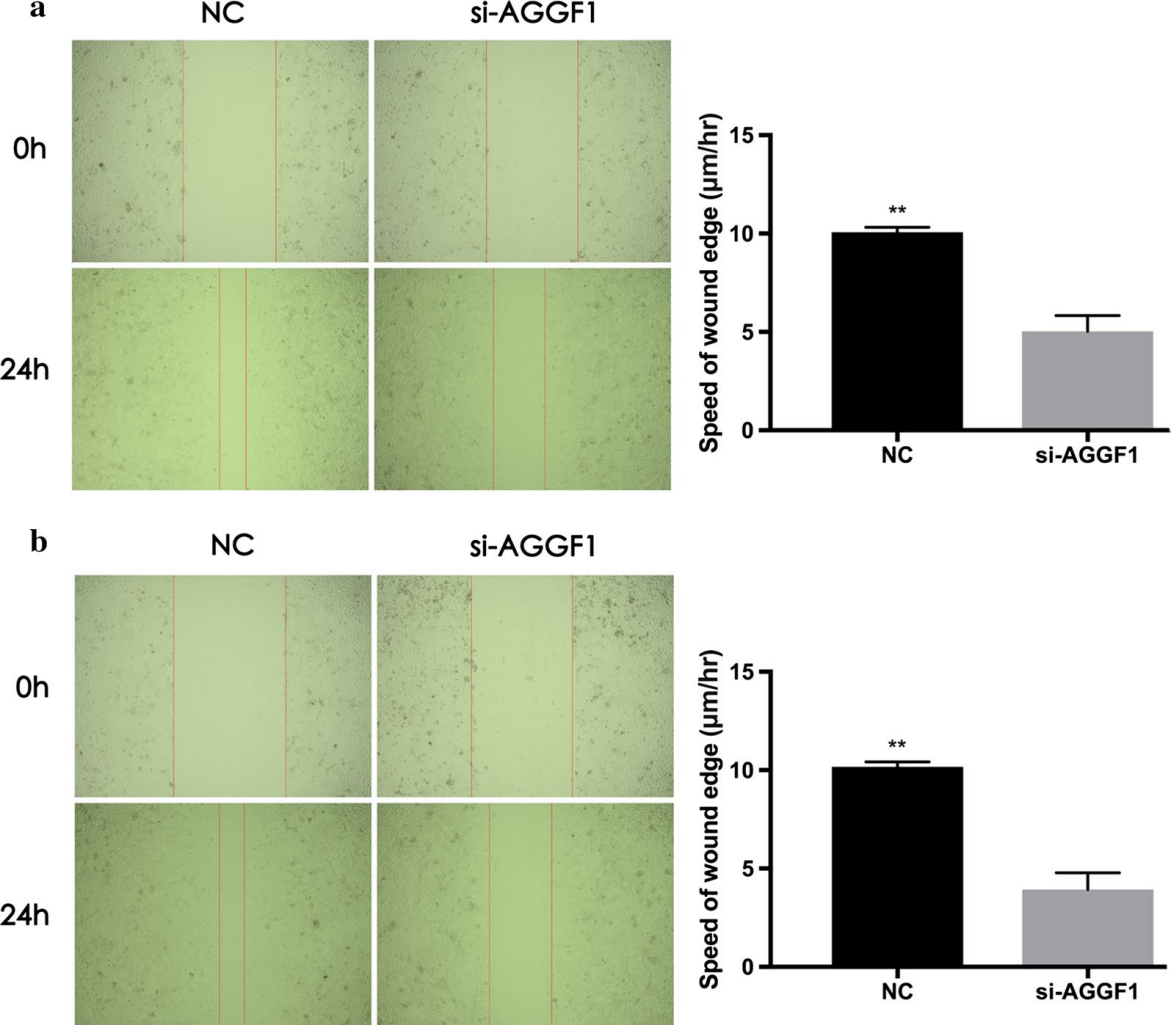
Representative images of wound-healing assays for MKN-45 cells (**a**) and MGC-803 cells (**b**) transfected si-NC or si-AGGF1 at 0 and 24 h post-transfection. Wound healing was quantified by measurement of the average linear speed of movement of the wound edges. All experiments were performed three times. Data were presented as mean ± SD. **P < 0.01

**Fig. 3:**
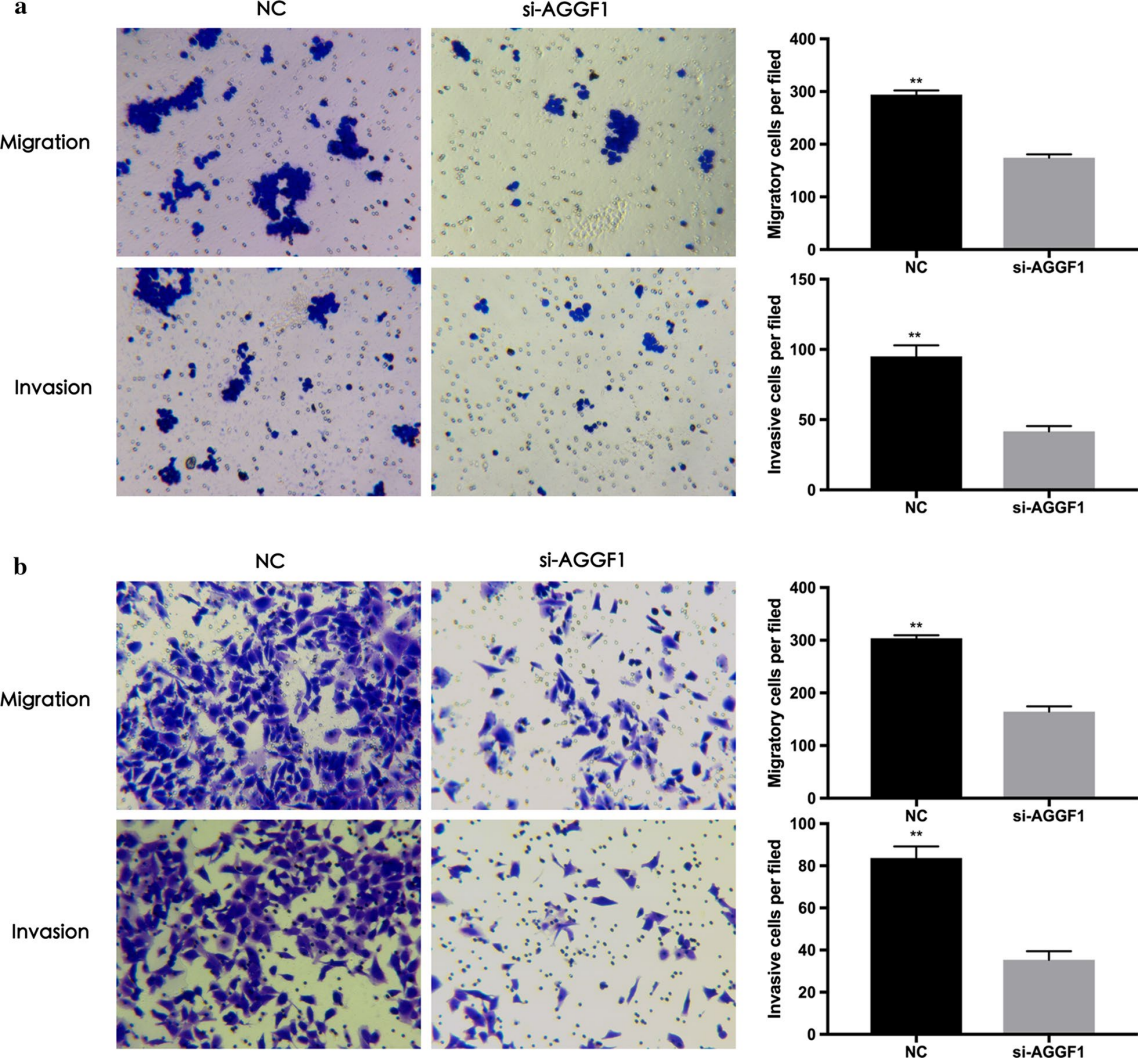
Representative images and bar graphs depicting the migration and invasion ability of MKN-45 cells (**a**) and MGC-803 cells (**b**) after si-NC or si-AGGF1 transfection (×100). Data were presented as mean ± SD. **P < 0.01

To further explore the mechanistic role of AGGF1 in GC, knockdown of AGGF1 gene expression by small RNA interfering method was performed in the MKN-45 and MGC-803 cell lines (Additional file 2: Figure S1). The results showed that MKN-45 and MGC-803 cells with AGGF1 knockdown had significantly slower closure of the wound area compared to their controls by wound-healing assay (both P < 0.01, Fig. 2). Besides, effects of ectopic AGGF1 expression on cell invasion and migration potential of MKN-45 and MGC-803 cells were also analyzed. In the transwell invasion and migration assay, cells with AGGF1 knockdown exhibited decreased invasion and migration abilities in MKN-45 (both P < 0.01, Fig. 3a) and MGC-803 cells (both P < 0.01, Fig. 3b) compared with those in the control groups.

### Knockdown of AGGF1 suppressed the tumor growth in an in vivo nude mice xenograft model

**Fig. 4:**
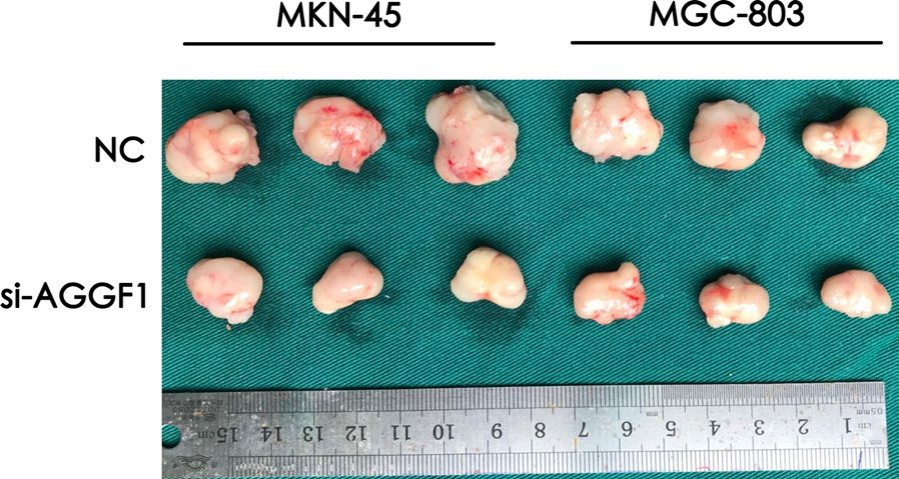
Representative images of excised tumors from five NOD/SCID mice at 40 days after injection with MKN-45 and MGC-803 cells transfected with sh-NC or sh-AGGF1

To evaluate the influence of AGGF1 knockdown on tumor growth, an in vivo nude mice xenograft model was established by using MKN-45 and MGC-803 cells. As shown in Fig. 4, both subcutaneous xenograft groups using transfected cells with sh-AGGF1 had dramatically decreased abilities to form tumors compared to those in control groups, as indicated by the final xenograft tumor size.

### Knockdown of AGGF1 inhibited the development of EMT in GC

**Fig. 5:**
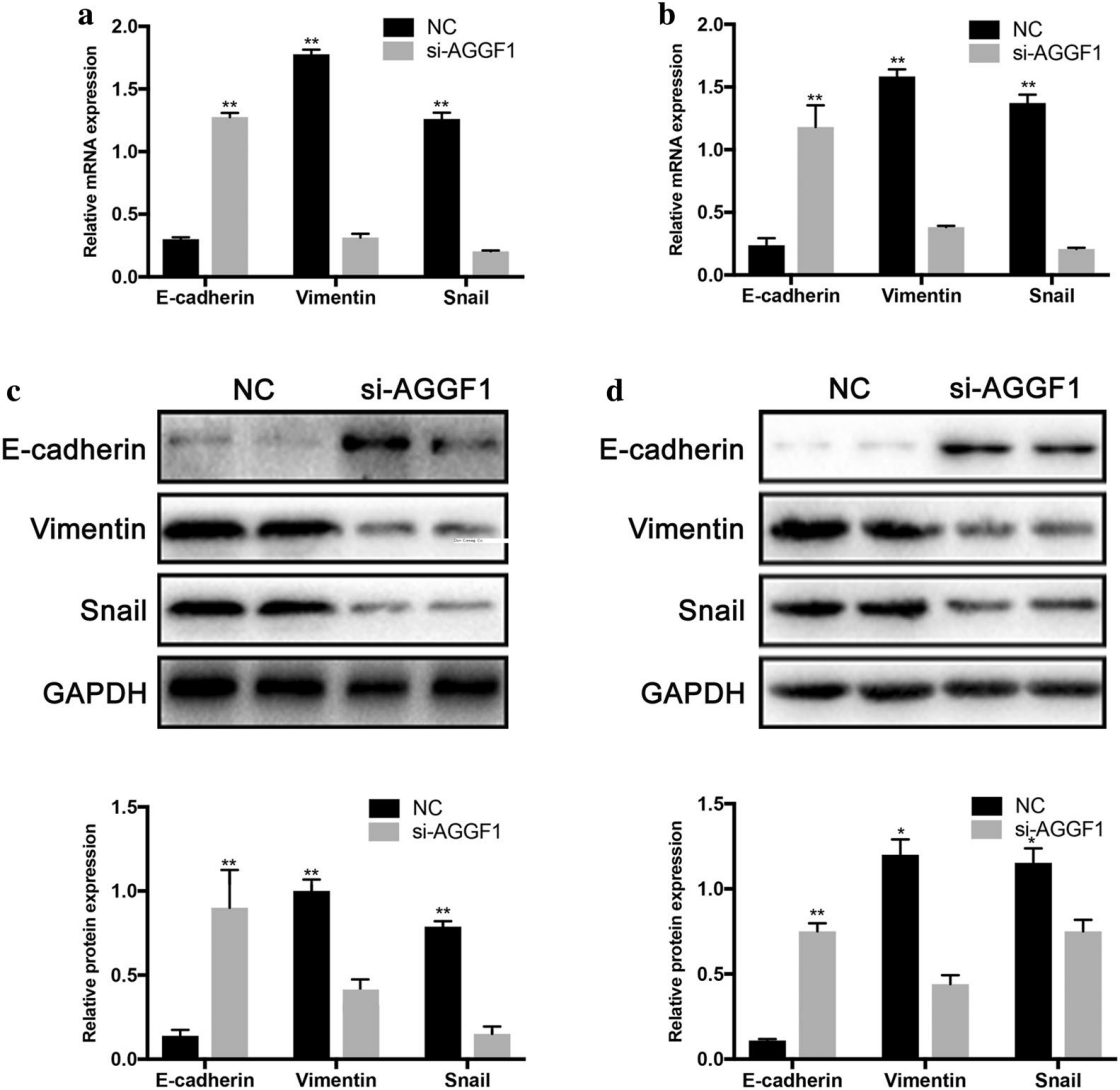
Relative mRNA and protein levels of epithelial and mesenchymal related markers in GC cells. Relative mRNA levels of epithelial and mesenchymal related markers in MKN-45 (**a**) and MGC-803 cells (**b**). Representative western blot gel documents and summarized data showing the protein levels of epithelial and mesenchymal markers in MKN-45 (**c**) and MGC-803 cells (**d**). *P < 0.05, **P < 0.01, versus NC group

To explore whether AGGF1 is associated with EMT, we examined the expression levels of epithelial and mesenchymal markers (E-cadherin, Vimentin, Snail) in the MKN-45 and MGC-803 cells with or without AGGF1 inhibition. The results showed that epithelial markers, E-cadherin, were increased compared with the controls, whereas mesenchymal marker Vimentin and related transcription factors Snail were significantly decreased in MKN-45 and MGC-803 cells with AGGF1 knockdown by qRT-PCR (Fig. 5a, b) and western blot (Fig. 5c, d) analyses. Consistently, gene silencing of AGGF1 retarded the development of EMT in MKN-45 and MGC-803 GC cells.

### AGGF1 regulated EMT by activating Wnt/β-catenin signaling pathway in GC

**Fig. 6:**
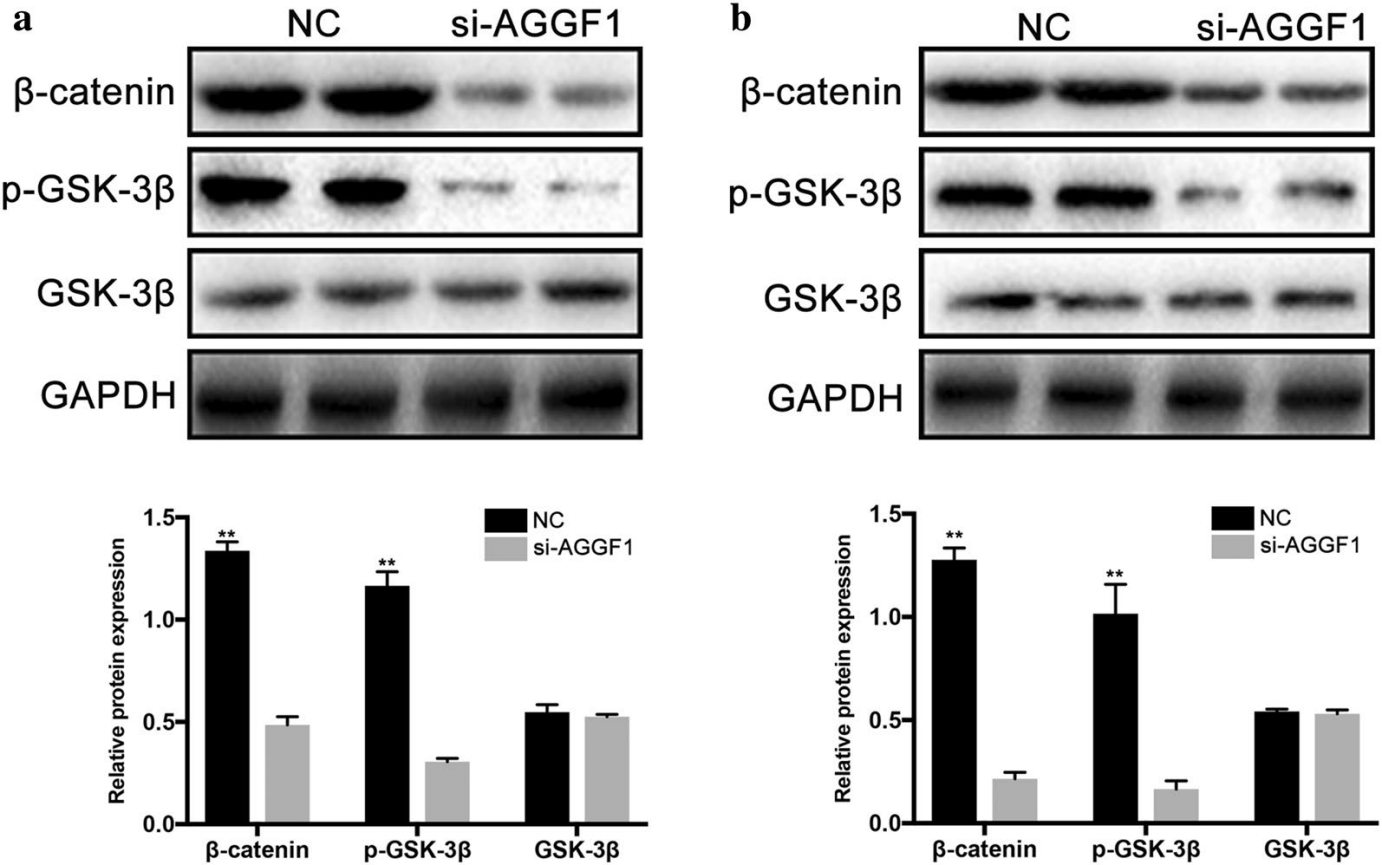
The role of AGGF1 in GC progression was mediated by activating Wnt/β-catenin signaling pathway. Representative western blot gel documents and summarized data showing the expression levels of total and phosphorylated GSK3β protein and β-catenin protein in MKN-45 (**a**) and MGC-803 (**b**) cells. **P < 0.01 versus NC group

**Fig. 7:**
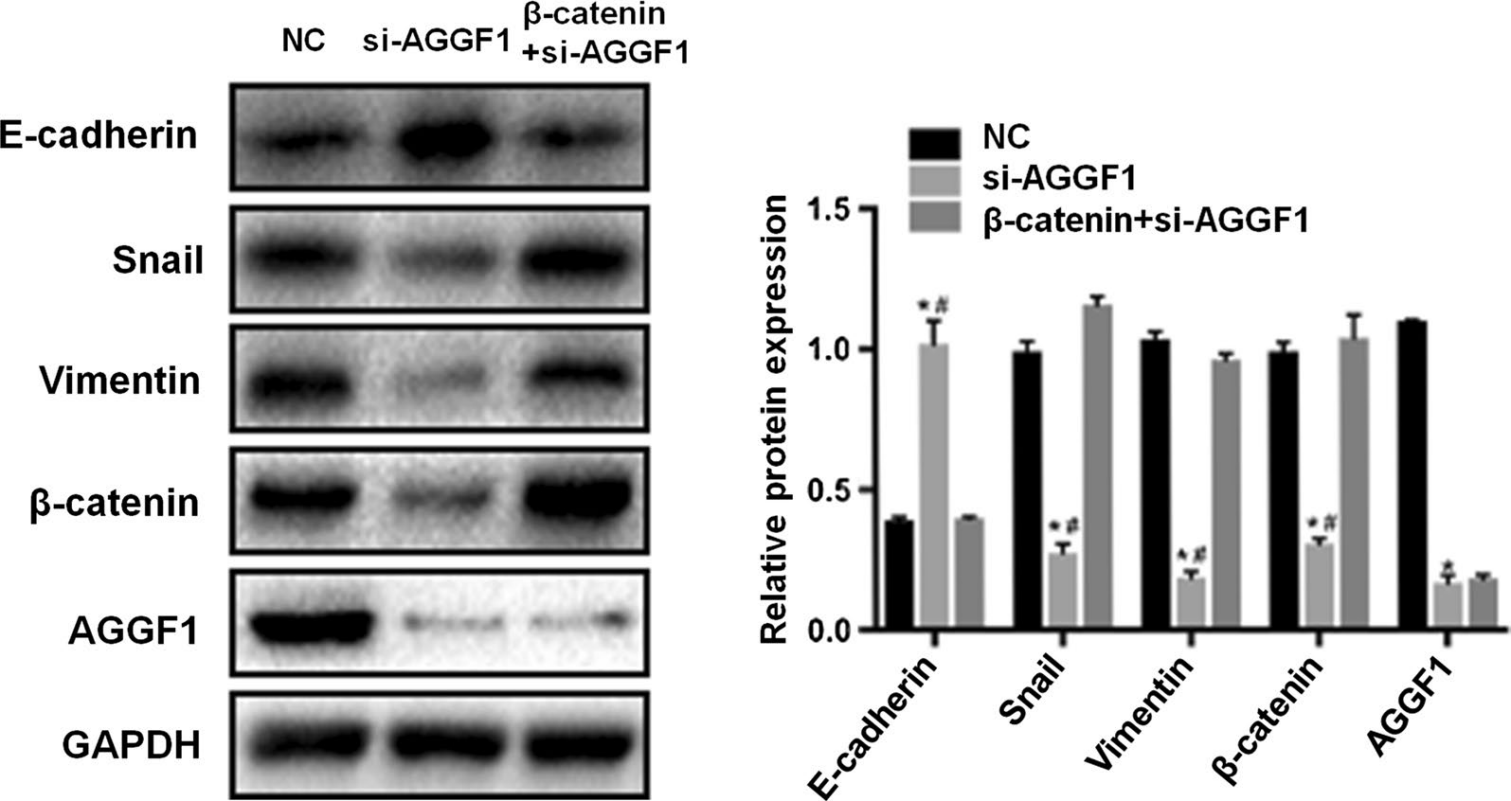
Overexpression of β-catenin counteracted the effects of AGGF1 on EMT. Representative western blot gel documents and summarized data showing the expression levels of E-cadherin, snail, vimentin, β-catenin and AGGF1 protein in MKN-45 cells transfected with NC, si-AGGF1 and β-catenin overexpression after si-AGGF1, respectively. *P < 0.05, versus NC group; ^#^P < 0.05 versus β-catenin overexpression after si-AGGF1 group

To evaluate the relationship between AGGF1 and Wnt/β-catenin signaling in GC, the activation status of GSK3β and β-catenin was firstly investigated. As shown in Fig. 6, the amount of phosphorylated GSK3β was significantly reduced by suppression of AGGF1 as well as the total β-catenin expression levels compared with control (all P < 0.01). To further demonstrate that Wnt/β-catenin acts as a critical pathway linking AGGF1 and EMT, we conducted the overexpression of β-catenin in the MKN-45 cells transfected with si-AGGF1. As shown in Fig. 7, E-cadherin level was increased together with decrease of snail, vimentin and β-catenin after AGGF1 knockdown in MKN-45 (all P < 0.05). Contrarily, while transfected with β-catenin plasmid in MKN-45 cell line with si-AGGF1, the above EMT markers showed a remarkable reversal (all P < 0.05).

## Discussion

Recently, several studies have found that AGGF1 was abnormally expressed or formed a novel AGGF1-PDGFRb fusion in malignancies [8–11, 17–19]. Besides, our previous study has revealed that AGGF1 expression was significantly associated with the lymph node metastasis, invasion depth and TNM stage of GC patients [12]. However, the potential molecular mechanisms underlying AGGF1 regulation in the metastasis and invasion of GC still remains unknown.

Therefore, in the present study, we performed in vitro and in vivo experiments to evaluate the influence of AGGF1 knockdown on the invasion and migration in GC, and explore its underlying mechanism. We firstly found that the expression of AGGF1 was significantly overexpressed both in GC tissues and cell lines. After knockdown of AGGF1 expression in GC cell lines, the results showed that invasion and migration of GC cell was dramatically retarded in vitro. Besides, AGGF1 knockdown group had smaller tumor xenografts than those in the control group in vivo. These results suggested that inhibition of AGGF1 significantly restrained GC tumorigenesis and invasion and migration.

Accumulating evidences have revealed that the migration and invasion of GC cells can be modulated by EMT mechanism [20–22]. Thus, to investigate whether AGGF1 is related to EMT, the expression levels of epithelial and mesenchymal markers (E-cadherin, Vimentin, Snail) were examined in the MKN-45 and MGC-803 cells with or without AGGF1 knockdown. The results showed that epithelial markers, E-cadherin, were increased compared with the controls, whereas mesenchymal marker Vimentin and related transcription factors Snail were significantly decreased in MKN-45 and MGC-803 cells with AGGF1 knockdown. These findings demonstrated that inhibition of AGGF1 could retard invasion and migration by suppressing EMT in GC.

Wnt/β-catenin signaling is one of the major pathways involved in EMT in the development and progression of many kinds of malignancies [22–25]. Previously, Major et al. [13] have found that AGGF1 associated with and regulated 40% of Wnt/β-catenin target genes and further raised the possibility that AGGF1 functionally contributed to Wnt/β-catenin signaling in colon cancer by integrative molecular screening. Thus, to evaluate the relationship between AGGF1 and Wnt/β-catenin signaling, we firstly investigated the activation status of GSK3β and β-catenin. The results showed that knockdown of AGGF1 attenuated Wnt/β-catenin related protein expression. The amount of phosphorylated GSK3β was significantly reduced by suppression of AGGF1 as well as the total β-catenin expression levels compared with control. Further, to demonstrate that Wnt/β-catenin acted as a critical pathway linking AGGF1 and EMT, we conducted the overexpression of β-catenin in MKN-45 with si-AGGF1. Western blot analysis confirmed the β-catenin overexpression could neutralize the effects of AGGF1 decrease on EMT as indicated by remarkable reversal of EMT molecular markers that had been changed. Collectively, these findings convincingly suggested AGGF1 could actuate the EMT through activating Wnt/β-catenin signaling in the development of GC.

However, some limitations of this study should be noted. First, immunochemical staining of AGGF1 wasn’t performed in the study due to our previously published results [12]. Second, only one siRNA or shRNA sequence of each target gene was used in the study and the experimental results might be interfered by the off-target effects to some extent. Third, protein–protein interaction experiments weren’t done, so the direct regulatory mechanism of AGGF1 involved in GC still remained unclear. All these above issues will be further explored in our future studies.

## Conclusions

The present study demonstrated that knockdown of AGGF1 could suppress oncogenic properties and inhibit migration and invasion of GC cells both in vitro and in vivo, the underlying mechanism of which was closely associated with the inhibition of EMT at least partially through Wnt/β-catenin pathway. The findings will provide the theoretical basis for recognizing AGGF1 as a potential therapeutic target in GC.

## Additional files

**Additional file 1: Table S1.** Characteristics of the 4 kinds of human gastric cancer cell lines used in the study.

**Additional file 2: Figure S1.** Knockdown of AGGF1 expression in both gastric cancer cell lines examined by western blot.
