# Supplementary material for: Knockdown of AGGF1 inhibits the invasion and migration of gastric cancer via epithelial–mesenchymal transition through Wnt/β-catenin pathway
Source: Cancer Cell Int. 2019 Feb 27;19:41. doi: 10.1186/s12935-019-0765-6 (PMC6391764; doi:10.1186/s12935-019-0765-6)
Supplement: Supplementary file 1 — Additional file 1: Table S1. Characteristics of the 4 kinds of human gastric cancer cell lines used in the study. [file 12935_2019_765_MOESM1_ESM.docx]

**Table S1:** Characteristics of the 4 kinds of human gastric cancer cell lines used in the study

| **Characteristics** | **SGC-7901** | **MKN-45** | **MGC-803** | **AGS** |
| --- | --- | --- | --- | --- |
| Age (years) | 56 | 62 | 53 | 54 |
| Gender | Female | Female | Male | Female |
| Tissue origin | Lymph node metastasis | Liver metastasis | Primary tumor | Primary tumor |
| Pathologic diagnosis | GAC | GAC | GAC | GAC |
| Histological classification | Poor | Poor | Poor | Middle |
| Country | P.R. China | Japan | P.R. China | USA |

Note: GAC: gastric adenocarcinoma
