# Supplementary figures and images for: Knockdown of AGGF1 inhibits the invasion and migration of gastric cancer via epithelial–mesenchymal transition through Wnt/β-catenin pathway
Source: Cancer Cell Int. 2019 Feb 27;19:41. doi: 10.1186/s12935-019-0765-6 (PMC6391764; doi:10.1186/s12935-019-0765-6)

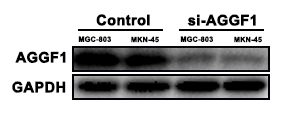

Supplement: Supplementary file 2 — Additional file 2: Figure S1. Knockdown of AGGF1 expression in both gastric cancer cell lines examined by western blot. [file 12935_2019_765_MOESM2_ESM.tif]
